# A Highly Sensitive Method for Quantitative Determination of L-Amino Acid Oxidase Activity Based on the Visualization of Ferric-Xylenol Orange Formation

**DOI:** 10.1371/journal.pone.0082483

**Published:** 2013-12-20

**Authors:** Zhiliang Yu, Ju Wang, Ning Zhou, Chuntian Zhao, Juanping Qiu

**Affiliations:** College of Biological and Environmental Engineering, Zhejiang University of Technology, Hangzhou, China; CNR, Italy

## Abstract

L-amino acid oxidase (LAAO) has important biological roles in many organisms, thus attracting great attention from researchers to establish its detection methods. In this study, a new quantitative in-gel determination of LAAO activity based on ferric-xylenol orange (Fe^III^XO) formation was established. This method showed that due to the conversion of Fe^II^ to Fe^III^ by H_2_O_2_ and subsequent formation of Fe^III^XO complex halo in agar medium, the logarithm of H_2_O_2_ concentration from 5 to 160 µM was linearly correlated to the diameter of purplish red Fe^III^XO halo. By extracting the LAAO-generated H_2_O_2_ concentration, the LAAO activity can be quantitatively determined. This Fe^III^XO agar assay is highly sensitive to detect H_2_O_2_ down to micromolar range. More importantly, it is easy to handle, cheap, reproducible, convenient and accurate. Coupled with SDS-PAGE, it can directly be used to determine the number and approximate molecular weight of LAAO in one assay. All these features make this in-gel Fe^III^XO assay useful and convenient as a general procedure for following enzyme purification, assaying fractions from a column, or observing changes in activity resulting from enzyme modifications, hence endowing this method with broad applications.

## Introduction

L-amino acid oxidases (LAAOs; EC 1.4.3.2) function in catalyzing the transformation of L-amino acids to the corresponding a-keto acids with the release of ammonium and hydrogen peroxide (H_2_O_2_) [1], [2]. Ever since the first discovery of LAAO from the bacterium *Proteus vulgaris* [3], LAAOs have been isolated from diverse organisms including snake venoms [4], insect drugs [5], sea hare [6], fungi [7], bacteria [8], [9] and algae [10]. LAAOs show broad biological activities including apoptosis, cytotoxicity, edema, hemolysis, hemorrhage, platelet aggregation, parasite-killing activity and antimicrobial activity, all of which are believed to be associated with the H_2_O_2_ production [11], [12]. LAAO activity has been characterized by quantifying the substances that are either consumed or generated in the redox reaction [13]–[15]. Among them, H_2_O_2_, one of the oxidative reaction products, is considered as an ideal substance for the detection of LAAO activity. The quantitative detection of H_2_O_2_ is mostly done by measuring the chemiluminescence due to the addition of horseradish peroxidase (HRP) and its substrate. There exists the commercially available kit (Amplex® Red Hydrogen Peroxide/Peroxidase Assay Kit, Invitrogen, USA), with detection limit down to micromolar level. [16]. However, most of the HRP substrates are mutagenic, carcinogenic or extremely toxic compounds, and HRP itself is easily inactivated and very expensive. Recently, we established a Prussian blue agar assay for quantitatively determining the LAAO activity [17]. In brief, iron (III) and potassium hexacyanoferrate (III) in the assay can be oxidized to yield the blue precipitate of Prussian blue where the H_2_O_2_ produced by LAAO activity acts as electron donor. Although the Prussian blue agar assay is cheaper and more convenient than the HRP-based assays, there are still several drawbacks that may limit its further application. Firstly, its quantitative detection limit is only down to about 0.5 mM level of H_2_O_2_. Secondly, potassium hexacyanoferrate (III) itself is safe, but under the peracidic condition it may degrade and release extremely toxic CN^−^. Thirdly, Prussian blue is a complicate class of chemical compounds containing Prussian blue, Prussian brown, Prussian white and Berlin green, which is sensitive to pH condition. Therefore, extremely careful pH adjustment in the Prussian agar preparation is required to produce reproducible result of color formation. It is desirable to develop an assay combining the advantages of HRP-based and Prussian blue-based measurement.

Like Prussian blue, xylenol orange (XO, 3, 3′-Bis[N,N-bis(carboxymethyl) aminomethyl]-o-cresolsulfonephthalein) is one of the most important color materials. Considering that ferrous ion (Fe^II^) can be oxidized to ferric ion (Fe^III^) in the presence of H_2_O_2_, previous studies have demonstrated that XO can be applied to measure H_2_O_2_ by spectrophotometrically analyzing the purplish red complex (ferric-xylenol orange, Fe^III^XO) that is formed by Fe^III^ and XO [18]. This XO-based assay in solution can detect down to micromolar level of H_2_O_2_ [18], thus in theory providing higher H_2_O_2_ sensitivity, compared to Prussian blue agar assay. The purpose of this study is to describe a new application of the Fe^III^XO formation for quantitatively determining the LAAO activity by in-gel visualization and measurement of H_2_O_2_. This new Fe^III^XO assay is not only comparative to the HRP-involved assay in terms of sensitivity, but also bears similar benefits of Prussian blue agar assay, including ease of handling and cost-effectiveness. Moreover, it can be directly used for in-gel determination of the number and molecular weight of LAAO on the SDS-PAGE after visualization of the purplish red Fe^III^XO complex.

## Results

Fe^III^XO complex formation can be used to detect the concentration of hydroperoxides [18]. Fe^II^ can be oxidized by H_2_O_2_ to Fe^III^ which will sequentially coordinate with XO to yield purplish red Fe^III^XO complex as shown below.
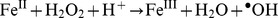


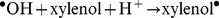


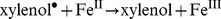


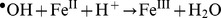


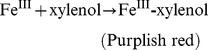
These reactions are fast, sensitive and reproducible. The concentration of H_2_O_2_ can usually be determined by spectrophotometrically measuring purplish red color of Fe^III^XO complex in solution [19]. In the present study, we, for the first time, tried to adapt the Fe^III^XO assay in agar gel to extracting the concentration of standard H_2_O_2_ or H_2_O_2_ produced by LAAO activity through directly measuring the diameter of the purplish red halo.

The formation of Fe^III^XO complex requires acidic condition where 25 mM H_2_SO_4_ is usually appropriate for pH capacity of spectrophotometrical Fe^III^XO assay in solution [20]. However, in our solid Fe^III^XO agar assay, 3–10 mM H_2_SO_4_ were proper for acidic condition and gave purplish red halos with almost saturated diameter driven by 40 µM H_2_O_2_ (Figure S1). No purplish red zone of Fe^III^XO was formed if the agar medium was devoid of H_2_SO_4_. On the other hand, further increase of H_2_SO_4_ concentration in assay agar resulted in the decrease of the size of purplish red halo. Since the color of purplish red zone of Fe^III^XO on assay agar with 3 mM H_2_SO_4_ was blurred (data not shown), the proper concentration of H_2_SO_4_ for Fe^III^XO agar assay is 6 mM up to 10 mM, giving final pH of 3.5 down to 2.3. After pouring, the Fe^II^XO agar plates consistently gave shallow orange red color as XO (pH indicator) itself will show orange red under acidic condition.

FeSO_4_ and XO are the two major elements in Fe^II^XO agar. To investigate the effect of the molar ratio of FeSO_4_ to XO on the Fe^III^XO agar assay, FeSO_4_ with different concentrations from 0 to 0.4 mM were added to assay medium while XO was fixed at 0.15 mM. As shown in Figure S2, at 40 µM H_2_O_2_ condition, the diameter of purplish red halo increased with higher concentration of FeSO_4_ and appeared to a maximum when the FeSO_4_ concentration reached 0.25 mM. Further increase of FeSO_4_ concentrations from 0.25 mM to 0.4 mM cannot obviously enlarge the purplish red zone, indicating that the Fe^III^XO was saturated when the molar ratio of FeSO_4_ to XO reached 5∶3.

**Figure 1:**
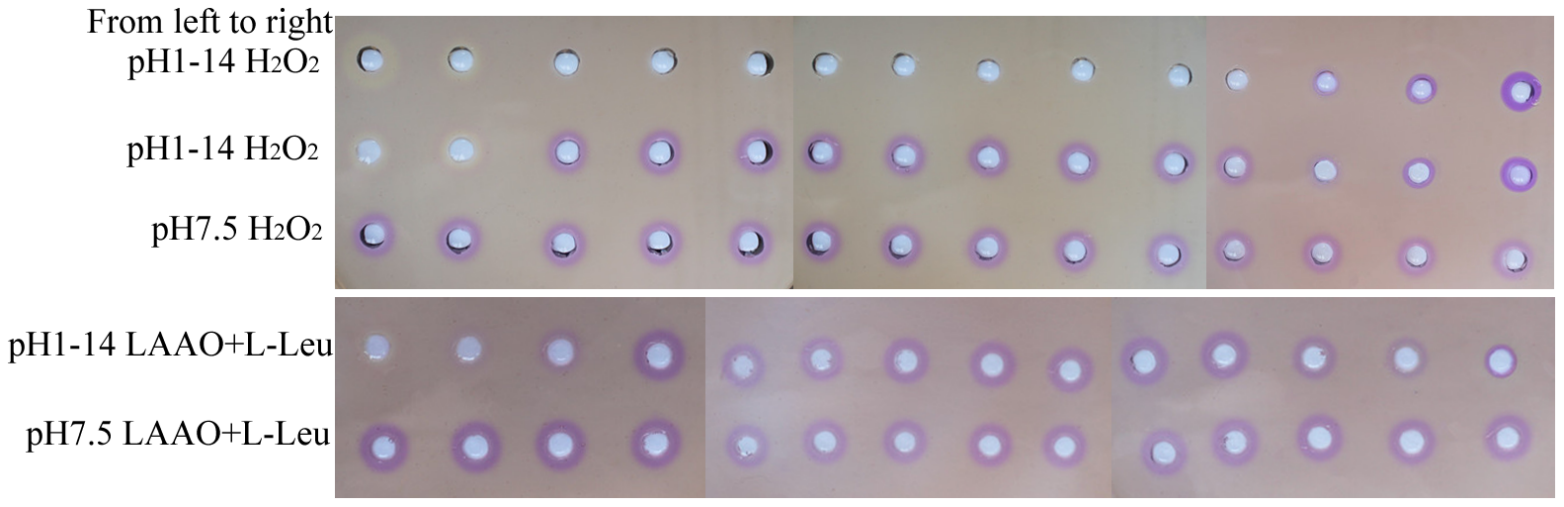
Dependence of detection solution pH for detection of standard H_2_O_2_ or H_2_O_2_ caused by LAAO activity in Fe^III^XO agar assay. Row 1: 14 background solutions with different pH values from 1 to 14 (from left to right) prepared by a mixture of 6 N HCl and 6 N NaOH; row 2: 14 standard 20 µM H_2_O_2_ solutions with different pH values ranging from 1 to 14 (from left to right) adjusted by either HCl or NaOH; row 3: 14 standard 20 µM H_2_O_2_ solutions with uniform pH of 7.5; row 4: 14 individual oxidization reactions of L-Leu by LAAO from *Psudoalteromonas* sp. R3 (R3-LAAO) with different pH values from 1 to 14 (from left to right) adjusted by either HCl or NaOH after oxidation; row 5: 14 individual oxidization reactions of L-Leu by R3-LAAO with uniform pH of 7.5.

Since Fe^II^XO agar medium was pH-sensitive, we also investigated the effect of pH on the color development of Fe^III^XO agar. The background solutions with different pH values ranging from 1 to 14 were prepared by mixing 6N HCl with 6N NaOH as required. As indicated in row 1 of Figure 1, the background solutions with pH from 3 to 11 did not cause noticeable color change of Fe^III^XO agar, remaining the original orange red. However, both lower pH and higher pH did make the color change of the agar medium. The background solutions with pH≤2 yielded lemon yellow halos, most likely due to the color presentation of XO as a pH indicator under peracidic condition. The lower the pH, the bigger and stronger the lemon yellow halos. On the other hand, when the background pH was above 12, the purplish red halos were generated even without H_2_O_2_ treatment (row 1 of Figure 1). There are two possible reasons: (1) as a pH indicator, XO will present a color of purplish red when pH≥12; (2) when pH≥12, Fe^II^ could easily be oxidized to Fe^III^ by the oxidant like oxygen and subsequently form purplish red Fe^III^XO with XO. The higher the pH from 12 to 14, the bigger and stronger the purplish red halos. Similarly, 20 µM H_2_O_2_ solutions with different pH values from 1 to 14 yielded different resultant halos (row 2 in Figure 1). Both the peracidic (pH≤2) and peralkaline (pH≥12) conditions had remarkable influence on the color development of assay agar under H_2_O_2_ pressure, while almost uniform sizes of purplish red halos were observed under 20 µM H_2_O_2_ with pH from 3 to 11. On the other hand, the 14 standard 20 µM H_2_O_2_ solutions with the same pH of 7.5 expectedly emerged consistent and reproducible purplish red halos with uniform size (row 3 in Figure 1). Similarly, the 14 oxidization reactions of L-Leu by LAAO from the LAAO-producer *Psudoalteromonas* sp. R3 (R3-LAAO) with the same pH of 7.5 also gave uniform and reproducible purplish red halos caused by the released H_2_O_2_ (row 5 in Figure 1). However, the oxidation solutions of L-Leu by R3-LAAO with different pH values adjusted to 1∼14 with HCl or NaOH after reactions yielded different resultant halos (row 4 in Figure 1). The pH values both ≤3 and ≥12 all obviously inhibited the formation of purplish red Fe^III^XO. In contrast, the adjusted pH ranging from 4 to 11 had no obvious inhibition. All these findings indicate that the proper pH of detection solution is 4 up to 11.

**Figure 2:**
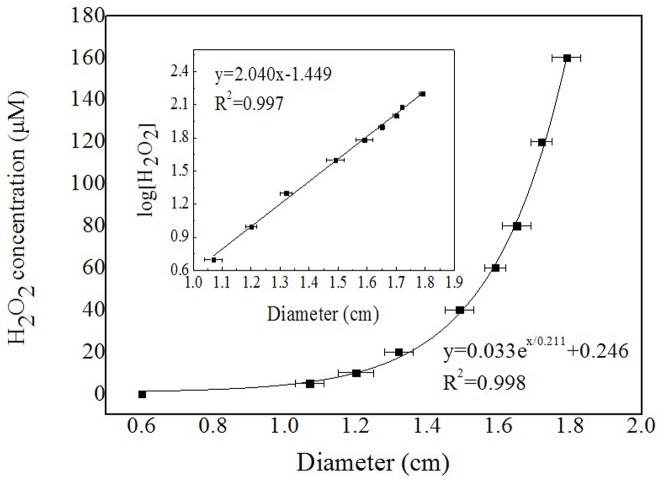
Correlation between the detected H_2_O_2_ concentration and the corresponding diameter of purplish red Fe^III^XO halo. The distribution can perfectly be fitted with an exponential equation y = 0.033e^×/0.241^+0.246 (R^2^ = 0.998), where x is the diameter of the purplish red halo and y the H_2_O_2_ concentration. Further plotting in inset displayed that the change in diameter of the purplish red halo was a function of logarithm of the H_2_O_2_ concentration in the range of 5 µM to 160 µM with linear fit under an equation y = 2.049×−1.460 (R^2^ = 0.997), where x is the diameter of the purplish red halo and y the logarithm of H_2_O_2_ concentration.

To quantify the LAAO activity, we first prepared a series of standard H_2_O_2_ solutions with different concentrations ranging from 0.5 µM to 250 µM with the same pH of 7.5 for the Fe^III^XO agar assay. As displayed in Table 1, all the tested H_2_O_2_ gave purplish red zones, but with different sizes. The higher the H_2_O_2_ concentrations from 0.5 µM to 160 µM, the bigger the diameters of purplish red halos, indicating that Fe^III^XO agar assay is extremely sensitive and the diameters of the purplish red halos are positively associated with H_2_O_2_ concentrations over 0.5 µM to 160 µM. However, further increase of H_2_O_2_ concentration to 200 µM and even up to 250 µM did not obviously make the purplish red halo bigger, probably due to the saturation of Fe^III^XO agar by H_2_O_2_. The statistical analysis of mean difference of the purplish red halo diameters under different H_2_O_2_ concentrations showed that the increase of the halo diameter was extremely significant (P<0.001) with the increase of H_2_O_2_ concentration from 0 to 160 µM, but not significant (P>0.05) with the further increase of H_2_O_2_ concentration from 160 µM to 250 µM (Supplementary Table S1). To extract the correlation between the H_2_O_2_ concentrations ranging from 0 to 160 µM and diameters of purplish red halos, the data in Table 1 were plotted as displayed in Figure 2. The distribution can be fitted with an exponential equation y = 0.033e^×/0.241^+0.246 (R^2^ = 0.998), where x is the diameter and y the H_2_O_2_ concentration. Further plotting in Figure 2 inset showed that the change in diameter of the halo was a function of logarithm of the H_2_O_2_ concentration in a range of 5 µM to 160 µM with linear fit under an equation y = 2.049×−1.460 (R^2^ = 0.997), where x is the diameter and y the logarithm of H_2_O_2_ concentration.

**Table:** Table 1. The diameters of the purplish red halos under different concentrations of H_2_O_2_.

H_2_O_2_ concentration (µM)	log[H_2_O_2_]	Diameter (cm)
5	0.70	1.07±0.03
10	1.00	1.20±0.02
20	1.30	1.32±0.02
40	1.60	1.49±0.03
60	1.78	1.59±0.03
80	1.90	1.65±0.01
120	2.08	1.72±0.03
160	2.20	1.79±0.01
200	2.30	1.79±0.01
250	2.40	1.79±0.01

**Figure 3:**
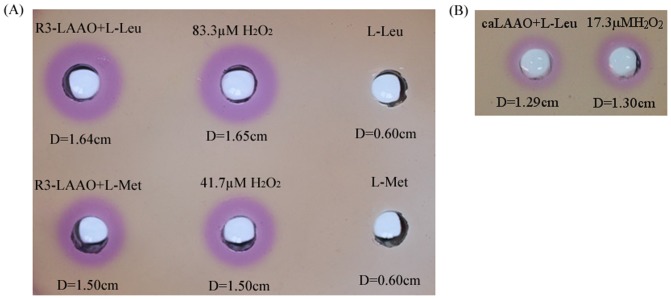
Characterization of LAAO activities from *Pseudoalteromonas* sp. R3 (R3-LAAO) with L-Leu and L-Met as substrates, respectively (A) and from *Crotalus adamanteus* venom (caLAAO) with L-Leu as substrate (B) based on the Fe^III^XO agar assay. On the basis of the diameters of the formed purplish red Fe^III^XO halos, the concentrations of H_2_O_2_ produced by LAAO activities were calculated with the equations in Figure 2. The corresponding standard H_2_O_2_ solutions as indicated above the corresponding holes were used to confirm the accuracy of Fe^III^XO agar assay. The diameters of the purplish red halos were marked below the holes. Without R3-LAAO, both L-Leu and L-Met (negative controls) did not give purplish red Fe^III^XO halos (right holes).

To evaluate the LAAO activity determined as H_2_O_2_ concentration fashion using the above extracted equation, R3-LAAO was used to oxidize the substrates L-Leu and L-Met, respectively, in separate reactions. After oxidization, the reaction solution was diluted 50 times and then subjected to Fe^III^XO agar assay. The results in Figure 3A showed that R3-LAAO with both L-Leu and L-Met as substrates can yield purplish red halos with diameters of 1.64 cm and 1.50 cm, respectively, which correspond to the H_2_O_2_ concentrations of 83.3 µM and 41.7 µM, respectively, on the basis of the above extracted equation in Figure 2. In contrast, without R3-LAAO, both L-Leu and L-Met (negative controls in right holes) did not give purplish red halos. To confirm the reliability of the extracted results, the standard H_2_O_2_ with concentrations of 83.3 µM and 41.7 µM were applied to Fe^III^XO agar assay and finally yielded the purplish red halos with diameters of 1.65 cm and 1.50 cm, respectively, all agreeing with our calculated concentrations. Therefore, the R3-LAAO activities with L-Leu and L-Met as substrates were 0.833 U/mL and 0.417 U/mL, respectively. All these findings indicate that the Fe^III^XO agar assay is feasible to sensitively detect the H_2_O_2_ produced by LAAO activity and the extracted equation is reliable to quantitatively determine the LAAO activity. To verify this method, another enzyme source, the commercial *Crotalus adamanteus* venom LAAO (caLAAO) was also used to oxidize L-Leu and applied to Fe^III^XO agar assay after 400 times dilution. Figure 3B showed that the oxidization solution of L-Leu by caLAAO gave a purplish red halo with 1.29 cm diameter which corresponds to 17.3 µM H_2_O_2_ based on the extracted equation. When 17.3 µM standard H_2_O_2_ was applied to Fe^III^XO agar assay, a purplish red zone with 1.30 cm diameter appeared. To further confirm its reliability, the oxidization solution of L-Leu by caLAAO was serially diluted by 100 times, 200 times, 300 times and 400 times, and subsequently subjected to Fe^III^XO agar assay. The results (Figure S3) showed that all detection solutions (in left holes) with different dilutions yielded purplish red halos with diameter of 1.60 cm, 1.46 cm, 1.38 cm and 1.30 cm, respectively, which correspond to the H_2_O_2_ concentration of 63.3 µM, 34.5 µM, 23.0 µM and 17.5 µM, respectively, representing the original H_2_O_2_ concentration in oxidization solution of 6.33 mM, 6.90 mM, 6.90 mM and 7.00 mM, respectively. As expected, the standard H_2_O_2_ (in right holes) with different concentrations of 63.3 µM, 34.5 µM, 23.0 µM and 17.5 µM gave purplish red halos with diameters of 1.60 cm, 1.46 cm, 1.38 cm and 1.30 cm, respectively, all agreeing with our calculated results. All these findings indicate that Fe^III^XO agar assay is very reliable for quantitatively detecting the LAAO activity.

**Figure 4:**
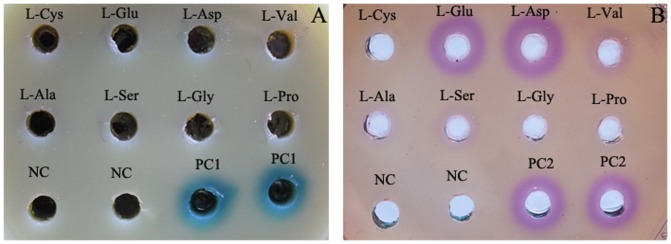
Comparison of detection sensitivity between Prussian blue agar assay (A) and Fe^III^XO agar assay (B). LAAO produced by *Pseudoalteromonas* sp. R3 (R3-LAAO) was used to oxidize the different L-amino acids including L-Cys, L-Glu, L-Asp, L-Val, L-Ala, L-Ser, L-Gly and L-Pro, in separate reactions, and subsequently the produced H_2_O_2_ in oxidization solution was measured with both Prussian blue agar assay [17] and Fe^III^XO agar assay. For the Prussian blue agar assay, the standard H_2_O_2_ with concentration of 10 mM was used as the positive control (PC1); in contrast, for the Fe^III^XO agar assay, the standard H_2_O_2_ with concentration of 50 µM was treated as the positive control (PC2). For both assays, the reaction solution without L-amino acid was used as the negative control (NC).

Compared with Prussian blue agar assay, whose quantitative detection limit of H_2_O_2_ concentration is around 500 µM [17], the Fe^III^XO agar assay is much more sensitive (around 100 times higher). To further compare their sensitivity, R3-LAAO was used to oxidize L-Cys, L-Glu, L-Asp, L-Val, L-Ala, L-Ser, L-Gly and L-Pro, in separate reactions, and subsequently the generated H_2_O_2_ was measured with both Prussian blue agar assays and Fe^III^XO agar assay. Results showed that no clear color was developed in Prussian blue agar plate (Figure 4A), suggesting that R3-LAAO has no obvious oxidization activity to those substrates. However, Fe^III^XO agar assay resulted in clear purplish red halos with L-Glu, L-Asp, L-Val, L-Ala and L-Ser as substrates (Figure 4B), indicating that R3-LAAO has activity to those substrates with an order of L-Asp>L-Glu>L-Val>L-Ser>L-Ala. All these findings indicate that the Fe^III^XO agar assay is much more sensitive than the Prussian blue agar assay.

Different methods have been developed to determine the LAAO number and its molecular weight [16], [17] since it is important for the characterization of the LAAO sequence. To achieve this purpose, Fe^III^XO agar assay was coupled with SDS-PAGE to determine the number and molecular weight of R3-LAAO. In brief, several replicates of R3-LAAO sample without heating to maintain its activity were electrophoresed on SDS-PAGE. After electrophoresis, different lanes of SDS-PAGE were sliced out for different treatments and subsequently put together on Fe^II^XO agar for color development. It was found in Figure S4 that the duplicate sample lanes (lanes 1 and 2) without Coomassie brilliant blue (CBB) staining clearly yielded a purplish red band on Fe^II^XO agar (below SDS-PAGE), indicating that R3-LAAO in SDS-PAGE bears only one active unit. Similarly, the two sample replicates (lanes 3 and 4) with CBB staining also gave a clear purplish red band at the same migration position on Fe^II^XO agar, revealing that R3-LAAO used in this assay is resistant to SDS and β-mercaptoethanol. As expected, the duplicate lanes 3 and 4 additionally showed CBB-stained protein bands in SDS-PAGE since R3-LAAO sample was precipitated from fermentation crude of *Psudoalteromonas* sp. R3 [17]. To determine which protein band bears the LAAO activity, all the CBB-stained protein bands near the purplish red band on Fe^II^XO agar were sliced out from a CBB-stained lane-4 replicate lane, and put on Fe^II^XO agar (lane 5). It was found that even after long time exposure to multiple steps treatment, the sliced target protein band with LAAO activity in SDS-PAGE still clearly yielded a purplish red band on Fe^II^XO agar, indicating that LAAO is very stable in this SDS-PAGE coupled Fe^III^XO agar assay. According to the molecular weights of CBB-stained standard protein marker (lane M), the corresponding protein band with LAAO activity from *Psudoalteromonas* sp. R3 was estimated to be around 65 kDa in size on SDS-PAGE, which is in agreement with the one achieved by SDS-PAGE coupled Prussian blue agar assay [17].

## Discussion

The diameters of the purplish red halos of Fe^III^XO complex driven by H_2_O_2_ is a linear function of logarithm of H_2_O_2_ concentration from 5 µM to 160 µM, thus allowing this assay to quantitatively determine the LAAO activity with a similar sensitivity as the HRP-involved methods. Although HRP is H_2_O_2_ sensitive probe, the assay is complex and expensive. Moreover, the solution of HRP is unstable and needs to be used right after it is ready. Besides, it needs detection instrument. In contrast, Fe^III^XO agar assay does not rely on any detection instrument, and it is simple, stable and cost-effective. Compared with Prussian blue agar assay [17], Fe^III^XO agar assay is more environmentally friendly. More importantly, it gives two orders of magnitude improvement in sensitivity (5 µM vs. 0.5 mM). Considering its high sensitivity and convenience, this Fe^III^XO agar assay can be used to differentiate the mutants with slight difference in LAAO activity from a mutant library with altered expression of LAAO, saving a great number of workload for the investigation of the involved regulation mechanisms underlying the LAAO production.

As reported [20], acidic condition is critical to the proper fabrication of Fe^II^XO agar before assay. Under non-acidic condition, the entire Fe^II^XO agar medium will soon become purplish red even in the absence of H_2_O_2_ (data not shown). Most probably, Fe^II^ in medium is unstable and will be easily oxidized by oxygen to Fe^III^ which will sequentially coordinate with XO to form purplish red Fe^III^XO complex. During the fabrication of Fe^II^XO agar, H_2_SO_4_ should be added to medium before FeSO_4_. Otherwise, the whole mixture of assay medium will also immediately turn to purplish red (data not shown). Another reason for acidic condition in Fe^II^XO agar medium is that it can help to avoid the hydrolysis of the iron [20]. Besides the fabrication of Fe^II^XO agar, the acidic capacity or low pH in agar medium is also required for the formation of Fe^III^XO complex driven by H_2_O_2_. As shown in Figure S1, no clear purplish red halo formation is observed on the Fe^II^XO agar treated with 40 µM H_2_O_2_ if if Fe^II^XO agar is lack of H_2_SO_4_ to give final pH 6. However, extreme acidic condition (pH 1.8 in the presence of 13 mM H_2_SO_4_) will make the F^II^XO agar medium become lemon yellow, and obscure the color development of Fe^III^XO complex. It is most likely that XO is responsible for the lemon yellow color of the agar medium under pH 1.8. Based on the above observation, a final pH value between 2.3 and 3.5 of the agar medium by supplying 6 mM to 10 mM H_2_SO_4_ is recommended for proper color development of the assay. We also show that the pH of the detection solution is crucial to the success of Fe^III^XO agar assay. Proper color development of Fe^III^XO agar is only observed when the detection solution has a pH between 4 and 11. When the pH is ≤2, XO itself will give strong lemon orange and thus mask the color development of purplish red Fe^III^XO complex caused by H_2_O_2_. When the pH is ≥12, there are two more sources responsible for the purplish red color development in addition to Fe^III^XO complex formation due to H_2_O_2_. First, XO itself will show purplish red color at this pH; second, under peralkaline condition, other oxidants, such as oxygen, can also easily convert Fe^II^ to Fe^III^, which will subsequently react with XO to form purplish red Fe^III^XO. Therefore, the detection of purplish red Fe^III^XO formed by H_2_O_2_ is not possible at pH above 12. Fortunately, the fermentation solutions from LAAO-producing microorganisms or direct LAAO enzymatic reaction solutions usually have pH values in the range of 4 to 11, thus giving this method broad applicability.

It has been reported that the complex Fe^III^XO has a 1∶1 stoichiometry [20]. However, in our method, the diameter of purplish red Fe^III^XO halo reaches equilibrium when the molar ratio of FeSO_4_ to XO in medium is close to 2∶1. XO and Fe^III^ can form Fe^III^_2_XO, Fe^III^XO and Fe^III^XO_2_ since XO is a bi-functional metallochromic reagent, mainly depending on the molar ratio of iron ion to XO [21]. The 1∶2 complex (Fe^III^XO_2_) will form if XO is in excess. When the molar ratio of iron to XO approaches to 1∶1, the 1∶1 complex (Fe^III^XO) becomes predominant. In contrast, Fe^III^_2_XO will predominate in the presence of excess iron, which is attributed to XO's two isolated iminodiacetate groups that can bind metal ions. This is in agreement with our observation.

Coupled with SDS-PAGE, Fe^III^XO agar assay can be directly used to determine the numbers and approximate molecular weights of LAAO protein in one assay, giving crucial advantages over conventional spectrophotometric or fluorometric measurement. Without heating, the LAAO used in this study can tolerate SDS and β-mercaptoethanol, and maintain its activity even after long time exposure to the CBB-staining procedure and de-staining solution with glacial acetic acid. It is clear that knowing exactly the numbers and molecular weights of LAAO can benefit further purification and characterization of this enzyme. In particular, the sliced target band with LAAO activity can directly be analyzed with different techniques, such as protein sequencing and LC-MS/MS analysis.

With agar in medium, our Fe^III^XO agar assay can be performed based on the visual measurement rather than the spectrophotometric or fluorometric colorimetry, thus providing it with broad advantages of simplicity and cost-effectiveness. To push the visual threshold detection for trace H_2_O_2_ caused by LAAO activity, adjustment of the acidic condition of Fe^II^XO agar and near 2∶1 molar ratio of FeSO_4_ to XO are highly necessary. Besides, the addition of D-sorbitol to Fe^II^XO agar medium can also increase the sensitivity of this Fe^III^XO agar assay [24]. Combining all the above conditions, this in-gel method serves ideally as a sensitive procedure for quantitative determination of LAAO activity in following enzyme purification, assaying fractions from a column, or observing changes in activity resulting from enzyme modifications.

## Materials and Methods

### Chemicals and reagents

All chemicals are of at least analytical grade and used without further purification. H_2_O_2_ was purchased from Shanghai Lingfeng Chemical Reagent CO., LTD (China). Xylenol orange [o-cresosulfonphthalein-3, 3′-bis (methyliminodiacetate) sodium salt] and ferrous sulfate were purchased from SANGON BIOTECH (Shanghai, China), and D-sorbitol supplied by Biosharp CO., LTD (China).

LAAO from the marine bacterial *Psudoalteromonas* sp. R3 (R3-LAAO) was harvested as reported [17]. LAAO solution from *Crotalus adamanteus* venom (caLAAO) was purchased from Worthington Biochemical Corporation, USA.

### Fe^III^XO agar assay

Unless otherwise stated, Fe^III^XO agar assay was performed as follows: (1) prepare solution of ferrous-XO (Fe^II^XO) with 0.25 mM FeSO_4_, 6 mM H_2_SO_4_, 0.15 mM XO, 0.1 mM D-sorbitol and 1.5% agar; (2) dissolve the mixture completely at 100°C for 5 min and pour into glass Petri dish to make agar plate; (3) make circular wells on agar plate with a hole puncher whose diameter is 6 mm; (4) add 50 µL detection solutions containing standard H_2_O_2_ or H_2_O_2_ produced by LAAO activity to each well and wait for 60 min at room temperature for color change; (5) visualize the Fe^III^XO formation and measure the size of purplish red halo.

### Stereospecific oxidation of amino acid by LAAO activity

The stereospecific oxidation reaction was performed with 10 mM of each amino acid in 10 mL of R3-LAAO solution harvested from *Psudoalteromonas* sp. R3 culture supernatant [17] or caLAAO solution (mixture of 1 µL caLAAO with 10 mL of 0.1 M PBS buffer with pH 7.5, Worthington Biochemical Corporation, USA). Unless otherwise described, the pH of reaction mixture was adjusted to about 7.5. The reaction mixture was incubated at 37°C for 30 min. After oxidation, 50 µL detection solutions were subjected to either Fe^III^XO agar assay with appropriate dilution or Prussian blue agar assay without dilution [17].

### Determination of LAAO activity using Fe^III^XO agar assay

Unless otherwise stated, the determination of the LAAO activity includes the following steps: (1) 50 µL standard H_2_O_2_ solutions with different concentrations ranging from 0.5 µM to 250 µM with uniform pH 7.5 were subjected to Fe^III^XO agar assay; (2) after assay, the diameters of the formed purplish red Fe^III^XO halos were measured and the correlation equations between H_2_O_2_ concentrations and purplish red halo diameters were extracted using Origin software; (3) 50 µL oxidization solutions of L-amino acid by LAAO were applied to Fe^III^XO agar assay and the diameters of the generated purplish red halo were recorded; (4) the concentration of H_2_O_2_ produced by LAAO activity was calculated based on the extracted correlation equations between H_2_O_2_ concentrations and purplish red halo diameters; (5) the LAAO activity was determined with the fashion of the produced H_2_O_2_ concentration. One unit (U) is defined as the amount of enzyme that catalyses the formation of 1 mM H_2_O_2_/h at 37°C.

### SDS-polyacrylamide gel (SDS-PAGE) coupled Fe^III^XO agar assay

Unless otherwise described, SDS-PAGE coupled Fe^III^XO agar assay consists of the following three steps: (1) SDS-PAGE electrophoresis. The several replicates of detection samples containing LAAO were mixed with 4-fold sample loading buffer (1.0 M Tris-HCl, pH 6.8, 10% SDS, 20% β-mercaptoethanol, 50% glycerol, 1% bromophenol blue). Without heating, 20 µL resultant mixtures were separately applied to each well of normal SDS-PAGE with 5% stacking gel and 12% separation gel, as described by Laemmli [22]. Gel was run at a constant current of 4 mA until the dye reached the end of the gel; (2) Fe^III^XO agar assay using LAAO-contained gel. After SDS-PAGE electrophoresis, the entire gel was washed once with distilled water and cut into two pieces. One piece was directly put on Fe^II^XO agar for the color change; the other with the sample replicates was first stained with Coomassie brilliant blue (CBB) [23] and then also put on Fe^II^XO agar aside for the color development after three times wash with distilled water; (3) targeting of the protein with LAAO activity. After visualization of purplish red band on Fe^II^XO agar (below SDS-PAGE), the protein bands directly above the formed purplish red band area were cut out from the sample replicate of CBB stained SDS-PAGE and put on Fe^II^XO agar to determine the target band with LAAO activity which caused the formation of purplish red. If necessary, the standard protein ladder was used for the determination of molecular weight of target LAAO.

## Supporting Information

Table S1 **Statistical analysis of dependent variable diameters of purplish red halos from H_2_O_2_ with different concentrations by ANOVA.**
(DOC)

Figure S1 **Effect of acidic condition derived from H_2_SO_4_ with different concentrations in Fe^II^XO agar medium on the formation of purplish red Fe^III^XO complex caused by 40 µM H_2_O_2_.**
(TIF)

Figure S2 **Effect of FeSO_4_ in Fe^II^XO agar medium on the formation of purplish red Fe^III^XO caused by 40 µM H_2_O_2_.** Xylenol orange (XO) was fixed at 0.15 mM.
(TIF)

Figure S3 **Reliability of Fe^III^XO agar assay for determination of** ***Crotalus adamanteus*** **LAAO (caLAAO) activity.** The oxidization solutions of L-Leu by caLAAO were serially diluted by 100 times, 200 times, 300 times and 400 times, and 50 µL diluted solutions were subjected to Fe^III^XO agar assay (left hole). On the basis of the diameters of the formed purplish red Fe^III^XO halos, the concentrations of H_2_O_2_ produced by LAAO activities were calculated with the equations in Figure 2. The corresponding standard H_2_O_2_ solutions (right hole) as indicated above the corresponding halos were used to confirm the accuracy of Fe^III^XO agar assay. The diameters of the purplish red halos were marked below the holes.
(TIF)

Figure S4 **SDS-PAGE coupled in-gel Fe^III^XO agar assay for the characterization of LAAO from** ***Pseudoalteromonas*** **sp. R3 (R3-LAAO).** After electrophoresis, different lanes of SDS-PAGE with replicated samples were sliced out for different treatments and subsequently put together on Fe^II^XO agar for color development. Lane M: standard protein marker stained with Coomassie brilliant blue (CBB); Lanes 1 and 2: duplicate LAAO samples from *Pseudoalteromonas* sp. R3 (R3-LAAO) [17] without CBB staining; lanes 3 and 4: two replicates of lane-1 and lane-2 with CBB staining; lane 5: the sliced protein bands from a lane-4 replicate as indicated by arrow directly above the formed purplish red band area. The results showed that R3-LAAO in SDS-PAGE had only one active protein band to form purplish red band on Fe^II^XO agar (below SDS-PAGE) and its molecular weight was around 65 kDa.
(TIF)
