# Supplementary material for: A Highly Sensitive Method for Quantitative Determination of L-Amino Acid Oxidase Activity Based on the Visualization of Ferric-Xylenol Orange Formation
Source: PLoS One. 2013 Dec 20;8(12):e82483. doi: 10.1371/journal.pone.0082483 (PMC3869696; doi:10.1371/journal.pone.0082483)
Supplement: Table S1 — Statistical analysis of dependent variable diameters of purplish red halos from H2O2 with different concentrations by ANOVA. (DOC) [file pone.0082483.s001.doc]

|  | (I)H_2_O_2_ concentration  (µM) | (J)H_2_O_2_  Concentration  (µM ) | Mean Difference  (I-J)^a^ | Std. Error | P | 95% Confidence Interval | |
| --- | --- | --- | --- | --- | --- | --- | --- |
|  |  |  |  |  |  | Lower Bound | Upper Bound |
| LSD | 5 | 10µM | -.13000*** | .01626 | .000 | -.1639 | -.0961 |
|  |  | 20µM | -.25000*** | .01626 | .000 | -.2839 | -.2161 |
|  |  | 40µM | -.42000*** | .01626 | .000 | -.4539 | -.3861 |
|  |  | 60µM | -.52000*** | .01626 | .000 | -.5539 | -.4861 |
|  |  | 80µM | -.58000*** | .01626 | .000 | -.6139 | -.5461 |
|  |  | 120µM | -.65000*** | .01626 | .000 | -.6839 | -.6161 |
|  |  | 160µM | -.72333*** | .01626 | .000 | -.7573 | -.6894 |
|  |  | 200µM | -.72000*** | .01626 | .000 | -.7539 | -.6861 |
|  |  | 250µM | -.72333*** | .01626 | .000 | -.7573 | -.6894 |
|  | 10µM | 5µM | .13000*** | .01626 | .000 | .0961 | .1639 |
|  |  | 20µM | -.12000*** | .01626 | .000 | -.1539 | -.0861 |
|  |  | 40µM | -.29000*** | .01626 | .000 | -.3239 | -.2561 |
|  |  | 60µM | -.39000*** | .01626 | .000 | -.4239 | -.3561 |
|  |  | 80µM | -.45000*** | .01626 | .000 | -.4839 | -.4161 |
|  |  | 120µM | -.52000*** | .01626 | .000 | -.5539 | -.4861 |
|  |  | 160µM | -.59333*** | .01626 | .000 | -.6273 | -.5594 |
|  |  | 200µM | -.59000*** | .01626 | .000 | -.6239 | -.5561 |
|  |  | 250µM | -.59333*** | .01626 | .000 | -.6273 | -.5594 |
|  | 20µM | 5µM | .25000*** | .01626 | .000 | .2161 | .2839 |
|  |  | 10µM | .12000*** | .01626 | .000 | .0861 | .1539 |
|  |  | 40µM | -.17000*** | .01626 | .000 | -.2039 | -.1361 |
|  |  | 60µM | -.27000*** | .01626 | .000 | -.3039 | -.2361 |
|  |  | 80µM | -.33000*** | .01626 | .000 | -.3639 | -.2961 |
|  |  | 120µM | -.40000*** | .01626 | .000 | -.4339 | -.3661 |
|  |  | 160µM | -.47333*** | .01626 | .000 | -.5073 | -.4394 |
|  |  | 200µM | -.47000*** | .01626 | .000 | -.5039 | -.4361 |
|  |  | 250µM | -.47333*** | .01626 | .000 | -.5073 | -.4394 |
|  | 40µM | 5µM | .42000*** | .01626 | .000 | .3861 | .4539 |
|  |  | 10µM | .29000*** | .01626 | .000 | .2561 | .3239 |
|  |  | 20µM | .17000*** | .01626 | .000 | .1361 | .2039 |
|  |  | 60µM | -.10000*** | .01626 | .000 | -.1339 | -.0661 |
|  |  | 80µM | -.16000*** | .01626 | .000 | -.1939 | -.1261 |
|  |  | 120µM | -.23000*** | .01626 | .000 | -.2639 | -.1961 |
|  |  | 160µM | -.30333*** | .01626 | .000 | -.3373 | -.2694 |
|  |  | 200µM | -.30000*** | .01626 | .000 | -.3339 | -.2661 |
|  |  | 250µM | -.30333*** | .01626 | .000 | -.3373 | -.2694 |
|  | 60µM | 5µM | .52000*** | .01626 | .000 | .4861 | .5539 |
|  |  | 10µM | .39000*** | .01626 | .000 | .3561 | .4239 |
|  |  | 20µM | .27000*** | .01626 | .000 | .2361 | .3039 |
|  |  | 40µM | .10000*** | .01626 | .000 | .0661 | .1339 |
|  |  | 80µM | -.06000*** | .01626 | .001 | -.0939 | -.0261 |
|  |  | 120µM | -.13000*** | .01626 | .000 | -.1639 | -.0961 |
|  |  | 160µM | -.20333*** | .01626 | .000 | -.2373 | -.1694 |
|  |  | 200µM | -.20000*** | .01626 | .000 | -.2339 | -.1661 |
|  |  | 250µM | -.20333*** | .01626 | .000 | -.2373 | -.1694 |
|  | 80µM | 5µM | .58000*** | .01626 | .000 | .5461 | .6139 |
|  |  | 10µM | .45000*** | .01626 | .000 | .4161 | .4839 |
|  |  | 20µM | .33000*** | .01626 | .000 | .2961 | .3639 |
|  |  | 40µM | .16000*** | .01626 | .000 | .1261 | .1939 |
|  |  | 60µM | .06000*** | .01626 | .001 | .0261 | .0939 |
|  |  | 120µM | -.07000*** | .01626 | .000 | -.1039 | -.0361 |
|  |  | 160µM | -.14333*** | .01626 | .000 | -.1773 | -.1094 |
|  |  | 200µM | -.14000*** | .01626 | .000 | -.1739 | -.1061 |
|  |  | 250µM | -.14333*** | .01626 | .000 | -.1773 | -.1094 |
|  | 120µM | 5µM | .65000*** | .01626 | .000 | .6161 | .6839 |
|  |  | 10µM | .52000*** | .01626 | .000 | .4861 | .5539 |
|  |  | 20µM | .40000*** | .01626 | .000 | .3661 | .4339 |
|  |  | 40µM | .23000*** | .01626 | .000 | .1961 | .2639 |
|  |  | 60µM | .13000*** | .01626 | .000 | .0961 | .1639 |
|  |  | 80µM | .07000*** | .01626 | .000 | .0361 | .1039 |
|  |  | 160µM | -.07333*** | .01626 | .000 | -.1073 | -.0394 |
|  |  | 200µM | -.07000*** | .01626 | .000 | -.1039 | -.0361 |
|  |  | 250µM | -.07333*** | .01626 | .000 | -.1073 | -.0394 |
|  | 160µM | 5µM | .72333*** | .01626 | .000 | .6894 | .7573 |
|  |  | 10µM | .59333*** | .01626 | .000 | .5594 | .6273 |
|  |  | 20µM | .47333*** | .01626 | .000 | .4394 | .5073 |
|  |  | 40µM | .30333*** | .01626 | .000 | .2694 | .3373 |
|  |  | 60µM | .20333*** | .01626 | .000 | .1694 | .2373 |
|  |  | 80µM | .14333*** | .01626 | .000 | .1094 | .1773 |
|  |  | 120µM | .07333*** | .01626 | .000 | .0394 | .1073 |
|  |  | 200µM | .00333 | .01626 | .840 | -.0306 | .0373 |
|  |  | 250µM | .00000 | .01626 | 1.000 | -.0339 | .0339 |
|  | 200µM | 5µM | .72000*** | .01626 | .000 | .6861 | .7539 |
|  |  | 10µM | .59000*** | .01626 | .000 | .5561 | .6239 |
|  |  | 20µM | .47000*** | .01626 | .000 | .4361 | .5039 |
|  |  | 40µM | .30000*** | .01626 | .000 | .2661 | .3339 |
|  |  | 60µM | .20000*** | .01626 | .000 | .1661 | .2339 |
|  |  | 80µM | .14000*** | .01626 | .000 | .1061 | .1739 |
|  |  | 120µM | .07000*** | .01626 | .000 | .0361 | .1039 |
|  |  | 160µM | -.00333 | .01626 | .840 | -.0373 | .0306 |
|  |  | 250µM | -.00333 | .01626 | .840 | -.0373 | .0306 |
|  | 250µM | 5µM | .72333*** | .01626 | .000 | .6894 | .7573 |
|  |  | 10µM | .59333*** | .01626 | .000 | .5594 | .6273 |
|  |  | 20µM | .47333*** | .01626 | .000 | .4394 | .5073 |
|  |  | 40µM | .30333*** | .01626 | .000 | .2694 | .3373 |
|  |  | 60µM | .20333*** | .01626 | .000 | .1694 | .2373 |
|  |  | 80µM | .14333*** | .01626 | .000 | .1094 | .1773 |
|  |  | 120µM | .07333*** | .01626 | .000 | .0394 | .1073 |
|  |  | 160µM | .00000 | .01626 | 1.000 | -.0339 | .0339 |
|  |  | 200µM | .00333 | .01626 | .840 | -.0306 | .0373 |

^a^The mean difference is not significant at P>0.05 and extremely significant (***) at P<0.001.
