# Supplementary figures and images for: A Highly Sensitive Method for Quantitative Determination of L-Amino Acid Oxidase Activity Based on the Visualization of Ferric-Xylenol Orange Formation
Source: PLoS One. 2013 Dec 20;8(12):e82483. doi: 10.1371/journal.pone.0082483 (PMC3869696; doi:10.1371/journal.pone.0082483)

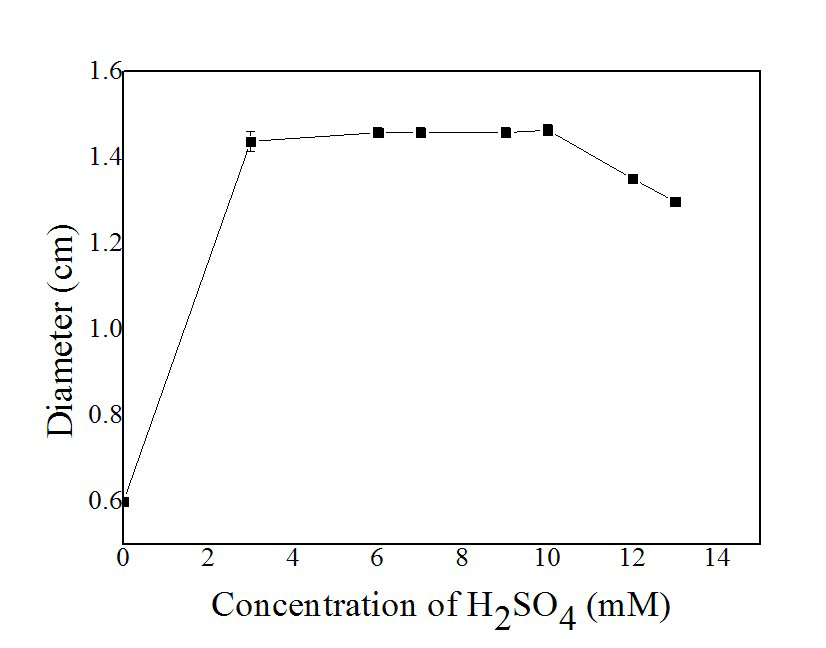

Supplement: Figure S1 — Effect of acidic condition derived from H2SO4 with different concentrations in FeIIXO agar medium on the formation of purplish red FeIIIXO complex caused by 40 µM H2O2. (TIF) [file pone.0082483.s002.tif]

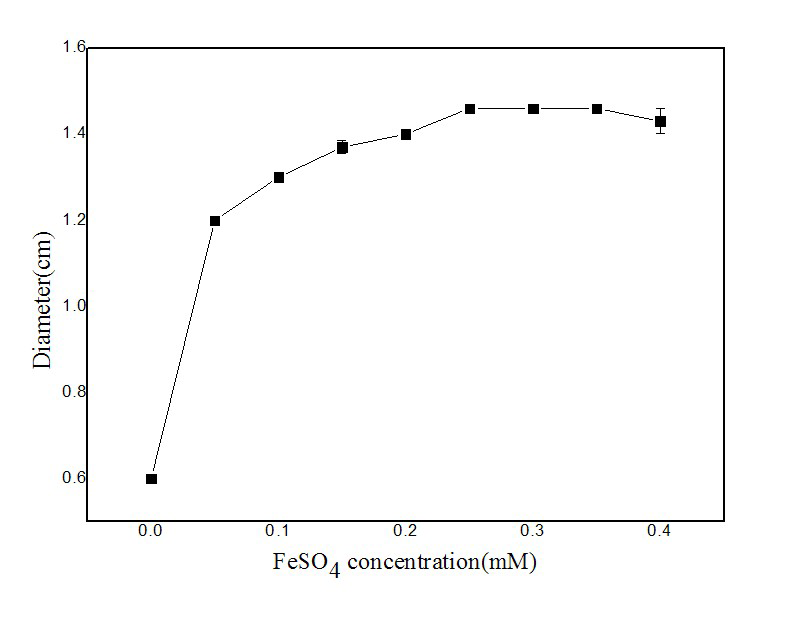

Supplement: Figure S2 — Effect of FeSO4 in FeIIXO agar medium on the formation of purplish red FeIIIXO caused by 40 µM H2O2. Xylenol orange (XO) was fixed at 0.15 mM. (TIF) [file pone.0082483.s003.tif]

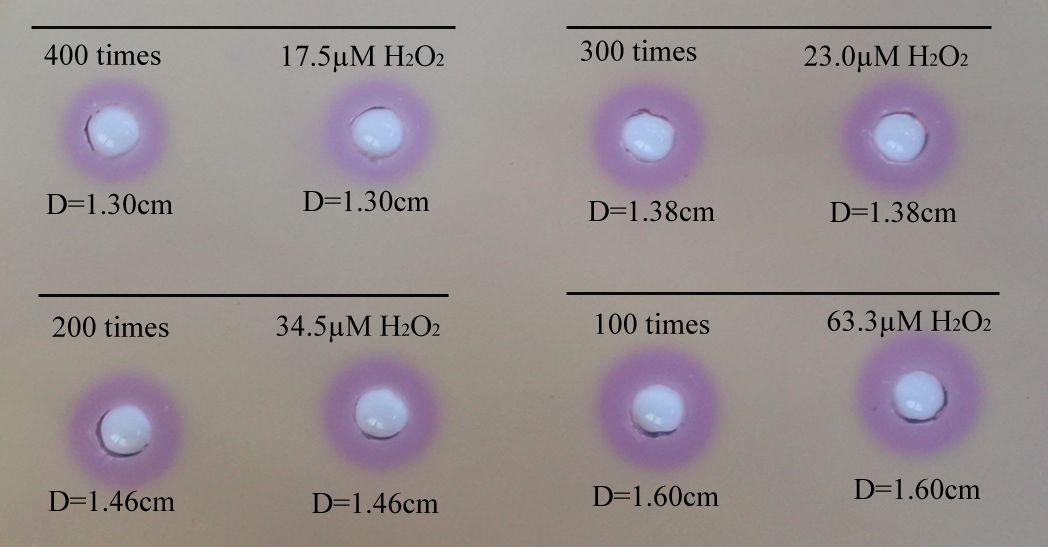

Supplement: Figure S3 — Reliability of FeIIIXO agar assay for determination of Crotalus adamanteus LAAO (caLAAO) activity. The oxidization solutions of L-Leu by caLAAO were serially diluted by 100 times, 200 times, 300 times and 400 times, and 50 µL diluted solutions were subjected to FeIIIXO agar assay (left hole). On the basis of the diameters of the formed purplish red FeIIIXO halos, the concentrations of H2O2 produced by LAAO activities were calculated with the equations in Figure 2. The corresponding standard H2O2 solutions (right hole) as indicated above the corresponding halos were used to confirm the accuracy of FeIIIXO agar assay. The diameters of the purplish red halos were marked below the holes. (TIF) [file pone.0082483.s004.tif]

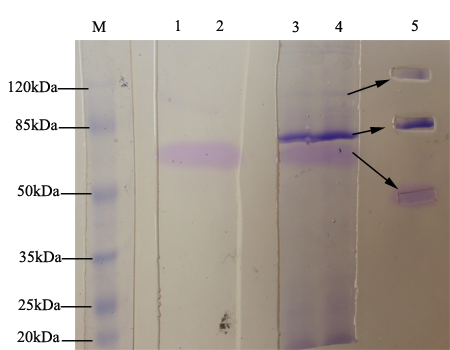

Supplement: Figure S4 — SDS-PAGE coupled in-gel FeIIIXO agar assay for the characterization of LAAO from Pseudoalteromonas sp. R3 (R3-LAAO). After electrophoresis, different lanes of SDS-PAGE with replicated samples were sliced out for different treatments and subsequently put together on FeIIXO agar for color development. Lane M: standard protein marker stained with Coomassie brilliant blue (CBB); Lanes 1 and 2: duplicate LAAO samples from Pseudoalteromonas sp. R3 (R3-LAAO) [17] without CBB staining; lanes 3 and 4: two replicates of lane-1 and lane-2 with CBB staining; lane 5: the sliced protein bands from a lane-4 replicate as indicated by arrow directly above the formed purplish red band area. The results showed that R3-LAAO in SDS-PAGE had only one active protein band to form purplish red band on FeIIXO agar (below SDS-PAGE) and its molecular weight was around 65 kDa. (TIF) [file pone.0082483.s005.tif]
